# U- and inverted U-shaped link between weight-adjusted waist index and chronic kidney disease in hyperuricemic adults

**DOI:** 10.1186/s12889-025-26076-8

**Published:** 2025-12-28

**Authors:** Danxuan Huang, Shuping Zhong

**Affiliations:** Department of Rheumatology, the Fifth Affiliated Hospital of Sun Yat-sen University, Zhuhai, People’s Republic of China

**Keywords:** Hyperuricemia, Weight-adjusted waist index (WWI), Chronic kidney disease (CKD), NHANES

## Abstract

**Background:**

Hyperuricemia is closely associated with an increased risk of chronic kidney disease (CKD) and is frequently accompanied by abnormal fat distribution. However, the relationship between fat distribution and CKD prevalence in hyperuricemic populations remains unclear. This study investigates the association between the weight-adjusted waist index (WWI) and CKD among adults with hyperuricemia, using data from the National Health and Nutrition Examination Survey (NHANES).

**Methods:**

Data from 5,330 hyperuricemic adults (2007–2018 NHANES) were analyzed. WWI was calculated by dividing waist circumference by the square root of body weight. CKD was defined by an estimated glomerular filtration rate (eGFR) falling below 60 mL/min/1.73 m² or by albuminuria levels greater than 30 mg/g. To evaluate the linkage between WWI and CKD, weighted multivariate logistic regression models and restricted cubic spline (RCS) analyses were utilized.

**Results:**

Individuals within the highest WWI quartile faced a 60% greater prevalence of CKD compared to the lowest quartile (OR = 1.60, 95% CI = 1.01–2.53, *p* = 0.047). RCS analysis revealed a composite pattern. The curve demonstrates a U-shape when WWI is below 11.36 cm/√kg, shifting to an inverted U-shape as WWI exceeds 11.36 cm/√kg.

**Conclusion:**

A significant association was observed between WWI and CKD prevalence in hyperuricemic adults, characterized by a composite U-shaped and inverted U-shaped relationship. This indicates the potential existence of an optimal WWI range associated with a lower prevalence of CKD. Further studies are required to confirm these observations.

**Supplementary Information:**

The online version contains supplementary material available at 10.1186/s12889-025-26076-8.

## Introduction

Hyperuricemia is a metabolic disorder caused by abnormalities in purine metabolism, where uric acid is the final product of purine nucleotide degradation in the body [1]. When the equilibrium between uric acid production and excretion is disrupted, this imbalance leads to the development of hyperuricemia. Globally, the prevalence of hyperuricemia ranges from 2.6% to 36%, with an upward trend observed across populations [2]. In the United States, the prevalence among adults was 14.6% in 2015–2016, affecting approximately 32.5 million people [3]. Chronic kidney disease (CKD) is a common complication of hyperuricemia. Long-term hyperuricemia significantly accelerates the decline in renal function by inducing oxidative stress, chronic inflammation, and endothelial dysfunction [4]. Moreover, hyperuricemia is significantly associated with increased kidney failure-related mortality, potentially shortening individuals’ life expectancy [5].

Hyperuricemia is not only closely related to kidney damage but also commonly accompanied by obesity and abnormal fat distribution. Elevated uric acid levels can influence fat generation and accumulation, promoting weight gain and central obesity [6]. Studies have shown that individuals with abnormal central fat distribution may face a higher risk of reduced glomerular filtration rate (GFR), which is linked to CKD development and progression [7]. Compared to healthy individuals, individuals with type 2 diabetes mellitus (T2DM) exhibit excessive fat accumulation, characterized by elevated visceral adipose tissue (VAT) levels that are negatively correlated with GFR. Reducing VAT may help slow CKD progression in T2DM individuals [8]. However, the relationship between fat distribution and CKD prevalence in hyperuricemic populations remains unclear and requires further investigation.

The weight-adjusted waist index (WWI) is an obesity indicator that combines body weight and waist circumference (WC) to better reflect central fat distribution. Unlike commonly used metrics like body mass index (BMI), WWI more accurately differentiates between muscle and fat mass, as well as central and peripheral fat, thereby providing a clearer link between fat distribution and adverse health outcomes. It has emerged as a promising tool for investigating the relationship between metabolic diseases and fat distribution [9]. Leveraging large-scale, nationally representative data from the National Health and Nutrition Examination Survey (NHANES), we examined the relationship between WWI and CKD in hyperuricemic individuals. This study aims to improve CKD prevalence identification in hyperuricemic individuals and provide scientific evidence for CKD management in this population.

## Methods

### Data source

The NHANES, conducted by the National Center for Health Statistics (NCHS), is a continuous, nationwide survey utilizing a multistage, complex sampling design to collect comprehensive data on demographics, dietary intake, physical examinations, laboratory assessments, and questionnaires. The survey assesses the health and nutritional status of both adult and pediatric populations in the United States. All NHANES protocols are approved by the NCHS Research Ethics Review Board. Further details are available on the official NHANES website (https://wwwn.cdc.gov/nchs/nhanes/).

### Study population

**Fig. 1:**
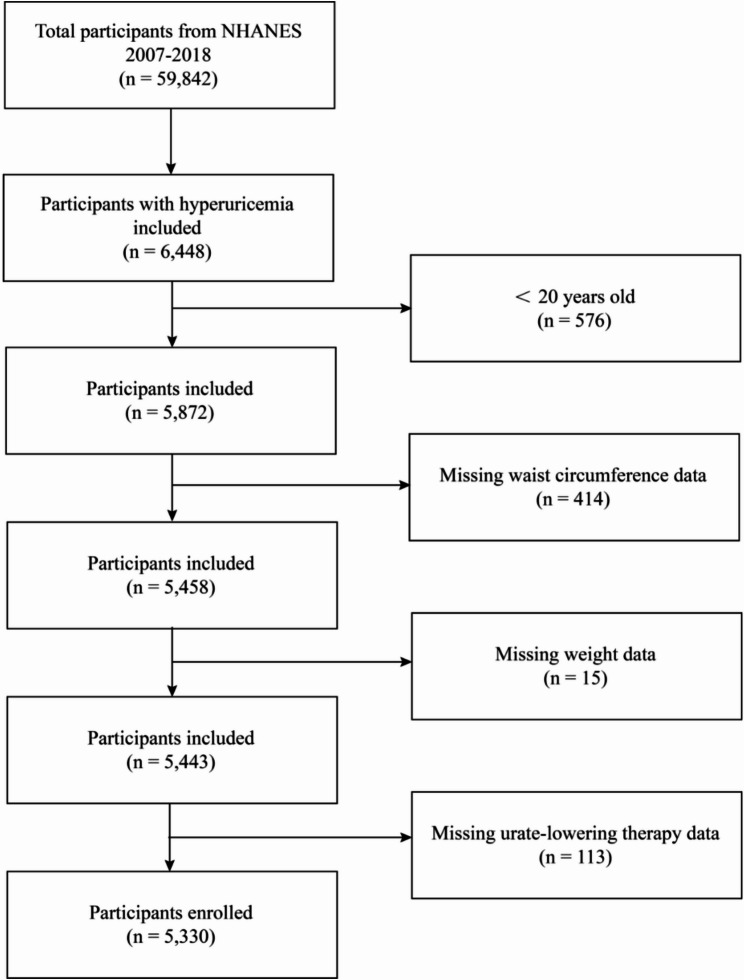
Flowchart of selecting study subjects from NHANES participants

This study utilized data from six NHANES cycles (2007–2018). Initially, 59,842 participants were included, and individuals diagnosed with hyperuricemia (*n* = 6,448) were selected as the study population. Exclusion criteria were as follows: (1) individuals younger than 20 years old (*n* = 576), (2) missing waist circumference data (*n* = 414), (3) missing weight data (*n* = 15), and (4) missing data on urate-lowering medication use (*n* = 113). Ultimately, a total of 5,330 participants were included in the study (Fig. 1).

### Weight-Adjusted Waist Index (WWI)

The WWI (cm/√kg) served as the exposure variable in this study, calculated by dividing WC (cm) by the square root of body weight (WT, kg) and rounded to two decimal places. WWI is positively correlated with central obesity. Trained health professionals measured WC and WT at mobile examination centers (MEC). Participants, dressed in standard MEC examination attire (disposable shirt, pants, and slippers), were weighed, while WC measurements were taken with participants crossing their arms over their chests, hands on opposite shoulders. Technicians, positioned on the participant’s right, identified the iliac crest and followed standardized measurement and recording protocols.

### Hyperuricemia and Chronic Kidney Disease (CKD)

The diagnostic criteria for hyperuricemia were defined as serum uric acid levels above 7 mg/dL in men and 6 mg/dL in women [10]. This definition is grounded in physiology: the 7 mg/dL threshold represents the established saturation point for urate crystallization, while the lower 6 mg/dL threshold for women accounts for their distinct physiology (e.g., estrogen’s uricosuric effect) to identify individuals with equivalent hyperuricemic risk [11, 12]. Uric acid levels were measured through uricase oxidation, producing a colorimetric product in reaction with peroxidase. CKD was defined according to KDIGO guidelines as an estimated glomerular filtration rate (eGFR) below 60 mL/min/1.73 m², albuminuria ≥ 30 mg/g, or both [13]. The eGFR, as an indicator of renal function, was calculated using the CKD-EPI equation based on serum creatinine [14]. Albuminuria was determined by the urine albumin-to-creatinine ratio. In NHANES, serum and urine creatinine levels were measured using the kinetic Jaffe method (2007–2016) and the enzymatic method (2017–2018), while urinary albumin concentrations were analyzed using a solid-phase fluorescence immunoassay technique.

### Covariates

(1) Demographic characteristics included age, race, gender, education level, and poverty income ratio (PIR). Age was categorized into older adults (≥ 65 years), middle-aged adults (40–64 years), and young adults (20–39 years). Race was classified as Mexican American, other Hispanic, non-Hispanic White, non-Hispanic Black, and other races. Educational attainment was divided into three groups: less than high school, high school or general education development (GED), and college or above. (2) Lifestyle factors included smoking status, defined as having smoked ≥ 100 or < 100 cigarettes in a lifetime. (3) Laboratory variables included serum calcium and phosphorus levels. (4) Health conditions included hypertension, diabetes, and hyperlipidemia. Hypertension was defined by a physician diagnosis, use of blood pressure-lowering medication, or an average systolic blood pressure ≥ 130 mmHg or diastolic ≥ 80 mmHg from three measurements. Diabetes was defined by a physician diagnosis, insulin or hypoglycemic medication use, hemoglobin A1c > 6.5%, or fasting blood glucose > 126 mg/dL. Hyperlipidemia was defined by a clinical diagnosis, use of lipid-lowering therapy, total cholesterol ≥ 240 mg/dL, HDL < 40 mg/dL for men or < 50 mg/dL for women, LDL ≥ 160 mg/dL, or triglycerides ≥ 150 mg/dL.

### Statistical analysis

The study population was divided into CKD and non-CKD groups, and baseline characteristics were compared. Normality tests indicated that continuous variables did not follow a normal distribution; thus, the Wilcoxon rank-sum test was applied for continuous variables, and the chi-square test was used for categorical variables. Continuous variables are presented as medians with interquartile ranges (25th and 75th percentiles), while categorical variables are shown as counts and percentages.

Weighted multivariate logistic regression analyses were conducted in three models to assess the independent association between WWI and CKD in hyperuricemic individuals. Results are presented as odds ratios (OR) with 95% confidence intervals (CI). Model 1 was unadjusted, Model 2 adjusted for demographic factors (age, gender, race), and Model 3 adjusted for all covariates, including demographic factors, education level, PIR, smoking status, blood pressure, blood glucose, blood lipid, serum calcium and phosphorus. Variance inflation factors were used to check for multicollinearity among variables.

Subgroup analyses were conducted with Bonferroni correction across strata defined by age, gender, blood pressure, blood glucose, and lipid levels to further explore the WWI-CKD association. Additionally, weighted restricted cubic spline analysis, combined with multivariate-adjusted logistic regression, was used to assess any nonlinear relationship between WWI and CKD. Statistical analyses were performed using R software (version 4.1.3).

## Results

### Baseline characteristics

The study included 5,330 participants aged 20 and older diagnosed with hyperuricemia, with an average age of 51 years. Among them, 43.95% were middle-aged, 56.24% were men, and 71.78% were non-Hispanic White. Participants were categorized based on the presence of CKD, with 22% diagnosed with CKD. Compared to the non-CKD group, individuals with CKD were generally older, with a higher proportion of females, non-Hispanic Whites, and smokers. The CKD group also showed higher rates of hypertension, diabetes, and hyperlipidemia, along with lower education and income levels. Serum calcium and phosphorus levels were similar between the two groups, with no significant differences. Moreover, WWI levels were higher in the CKD group (Table 1).

**Table 1:** Baseline characteristics of participants with Hyperuricemia, NHANES 2007–2018 (*N* = 5,330)

Characteristic	Overall, *N* = 5,330^1^	Non-CKD, *N* = 3,915 (78%)^1^	CKD, *N* = 1,415 (22%)^1^	*p*-value^2^
Age (years)	51 (35, 65)	47 (33, 60)	66 (53, 77)	< 0.001
Age				< 0.001
Elderly	1,718 (25.25%)	911 (16.57%)	807 (55.25%)	
Middle-aged	2,247 (43.95%)	1,768 (47.25%)	479 (32.57%)	
Younger adults	1,365 (30.80%)	1,236 (36.19%)	129 (12.18%)	
Gender				< 0.001
female	2,398 (43.76%)	1,654 (40.33%)	744 (55.64%)	
male	2,932 (56.24%)	2,261 (59.67%)	671 (44.36%)	
Race				0.041
Other Hispanic	279 (5.23%)	195 (5.55%)	84 (4.40%)	
Non-Hispanic White	1,299 (71.78%)	834 (71.28%)	465 (73.03%)	
Non-Hispanic Black	916 (13.52%)	599 (12.94%)	317 (15.01%)	
Other race	546 (9.47%)	427 (10.23%)	119 (7.55%)	
Education				< 0.001
less than high school	1,262 (15.58%)	833 (14.00%)	429 (21.06%)	
high school graduate/GED	1,300 (25.02%)	955 (24.73%)	345 (26.03%)	
some college or above	2,768 (59.40%)	2,127 (61.27%)	641 (52.91%)	
PIR				< 0.001
high income	2,450 (64.56%)	1,889 (66.71%)	561 (57.07%)	
middle income	1,366 (21.32%)	977 (20.26%)	389 (24.99%)	
low income	1,016 (14.12%)	700 (13.02%)	316 (17.94%)	
Smoke				0.016
Smoker	2,557 (47.30%)	1,824 (46.09%)	733 (51.48%)	
Non-smoker	2,773 (52.70%)	2,091 (53.91%)	682 (48.52%)	
Pressure				< 0.001
Hypertension	3,868 (68.42%)	2,610 (63.10%)	1,258 (86.80%)	
Non-hypertension	1,462 (31.58%)	1,305 (36.90%)	157 (13.20%)	
Glucose				< 0.001
Hyperglycemia	1,366 (19.09%)	753 (14.06%)	613 (36.49%)	
Non-Hyperglycemia	3,964 (80.91%)	3,162 (85.94%)	802 (63.51%)	
Lipids				< 0.001
Hyperlipidemia	3,902 (71.61%)	2,764 (69.35%)	1,138 (79.42%)	
Non-Hyperlipidemia	1,428 (28.39%)	1,151 (30.65%)	277 (20.58%)	
Calcium (8.5–10.5 mg/dL)	9.40 (9.20, 9.70)	9.50 (9.20, 9.70)	9.40 (9.20, 9.70)	0.101
Phosphorus (2.5–4.5 mg/dL)	3.70 (3.40, 4.10)	3.70 (3.40, 4.10)	3.70 (3.30, 4.10)	0.828
WWI (cm/√kg)	11.27 (10.72, 11.84)	11.14 (10.61, 11.70)	11.69 (11.17, 12.17)	< 0.001

^1^median (IQR) for continuous; *n* (%) for categorical

^2^Wilcoxon rank-sum test for complex survey samples; chi-squared test with Rao & Scott’s second-order correction

### Association between WWI and CKD in hyperuricemic populations

The association between WWI and CKD was assessed across three regression models (Table 2). In Model 1 (unadjusted) and Model 2 (adjusted for age, gender, and race), WWI was significantly positively associated with CKD. In Model 3 (adjusted for all covariates), the positive association between WWI and CKD was no longer significant (OR = 1.21, 95% CI = 0.98–1.50, *p* = 0.069). WWI was then categorized into quartiles: Q1 (8.51–10.72 cm/√kg), Q2 (10.72–11.27 cm/√kg), Q3 (11.27–11.84 cm/√kg), and Q4 (11.84–14.40 cm/√kg). In Model 2, participants in the highest WWI quartile (Q4) had a 2.15 times greater probability of CKD compared to the lowest quartile (Q1) (OR = 2.15, 95% CI = 1.38–3.35, *p* = 0.001). This positive association persisted in Model 3, where participants in the highest quartile (Q4) had a 60% increased prevalence of CKD compared to those in the lowest quartile (Q1) (OR = 1.60, 95% CI = 1.01–2.53, *p* = 0.047).

**Table 2:** Multivariable logistic regression analysis of the association between WWI and CKD in hyperuricemic populations

	Model 1		Model 2		Model 3	
Characteristic	OR (95% CI)	*p*-value	OR (95% CI)	*p*-value	OR (95% CI)	*p*-value
WWI (cm/√kg)	2.19 (1.96, 2.45)	< 0.001	1.48 (1.22, 1.81)	< 0.001	1.21 (0.98, 1.50)	0.069
WWI (quartile)						
Q1	1.00 (Reference)		1.00 (Reference)		1.00 (Reference)	
Q2	2.01 (1.55, 2.60)	< 0.001	1.38 (0.97, 1.97)	0.074	1.31 (0.88, 1.95)	0.184
Q3	3.12 (2.35, 4.14)	< 0.001	1.51 (0.95, 2.38)	0.078	1.36 (0.82, 2.25)	0.225
Q4	5.27 (4.03, 6.90)	< 0.001	2.15 (1.38, 3.35)	0.001	1.60 (1.01, 2.53)	0.047

Model 1: No adjustments for confounding factors. Model 2: Adjusted for age, gender, and race. Model 3: Adjusted for age, gender, race, education level, PIR, smoking status, blood pressure, blood glucose, blood lipid, serum calcium, and phosphorus levels. A *p*-value less than 0.05 indicates statistical significance

### Subgroup analysis

**Fig. 2:**
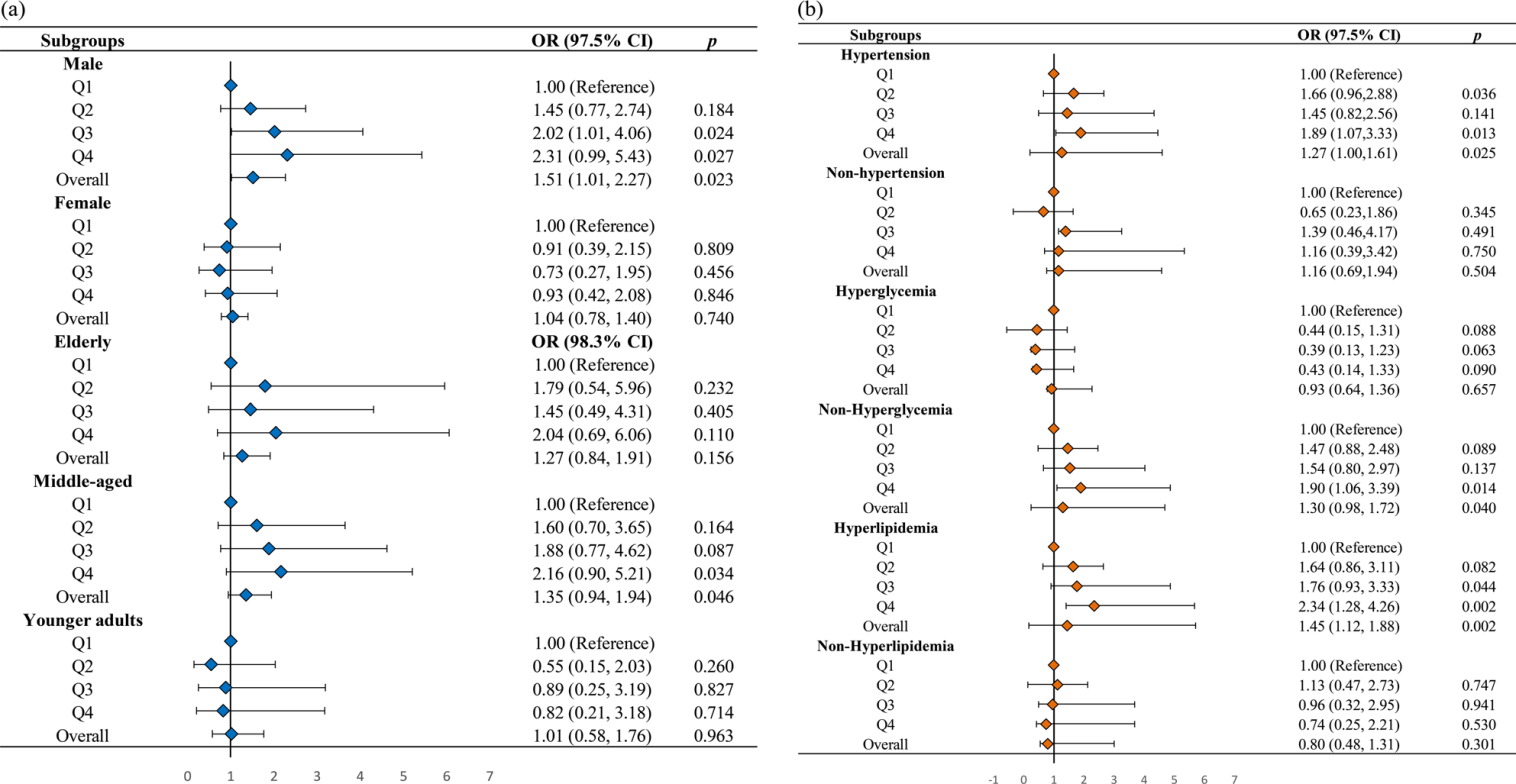
Subgroup analysis of the association between WWI and CKD in hyperuricemic populations (Model 3). The Model 3 is adjusted for age, gender, race, education level, PIR, smoking status, blood pressure, blood glucose, blood lipid, serum calcium, and phosphorus levels. Adjusted associations between WWI and CKD among individuals with hyperuricemia in selected subgroups. **a** Gender and age subgroups. **b** Blood pressure, blood glucose, and lipid subgroups

To further investigate the association between WWI and CKD in subpopulations with hyperuricemia, subgroup analyses were performed by gender, age, blood pressure, blood glucose, and lipid levels (Fig. 2). A significant positive association between WWI and CKD was observed in males and hyperlipidemia subgroups. In these two subgroups, participants in the highest WWI quartile (Q4) exhibited a significantly higher prevalence of CKD than those in the lowest quartile (Q1) (all Bonferroni-corrected *p* < 0.025). Additionally, a positive association between elevated WWI and increased CKD prevalence was also observed in hypertensive participants (Q4) and non-diabetic individuals (Q4) (*p* < 0.025).

### Restricted cubic spline (RCS) analysis

**Fig. 3:**
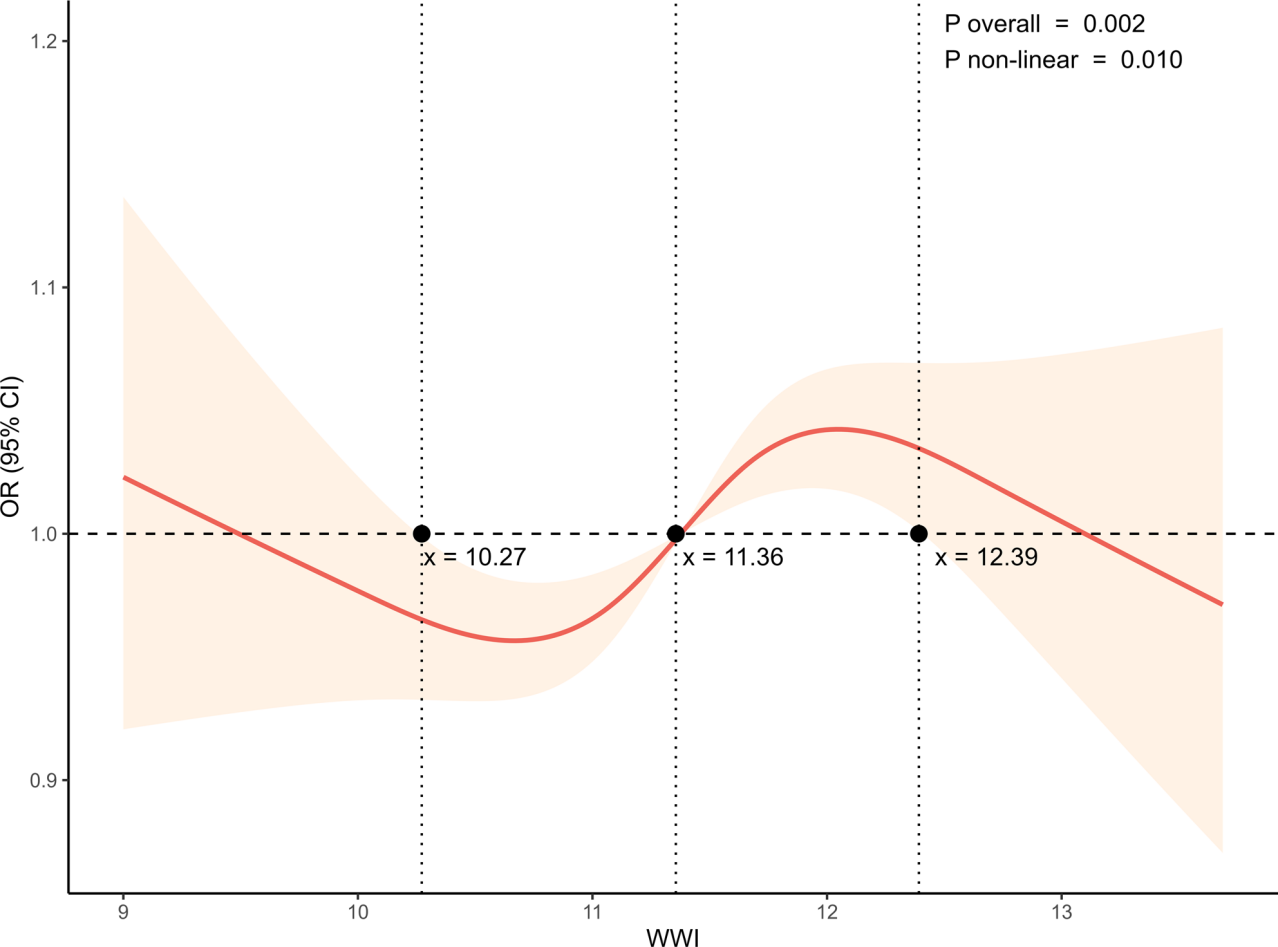
RCS analysis of the relationship between WWI and CKD in hyperuricemic populations (Model 3). The nonlinear association between WWI and CKD is shown, with the x-axis representing WWI and the y-axis representing the OR of CKD with corresponding 95% CI. The horizontal dashed line indicates OR = 1. A *p*-value less than 0.05 indicates statistical significance. Adjusted for age, gender, race, education level, PIR, smoking status, hypertension, diabetes, hyperlipidemia, serum calcium, and phosphorus levels (Model 3)

RCS analysis was used across various models to explore the dose-response relationship between WWI and CKD prevalence in hyperuricemic populations (Fig. 3; Fig.S1). All three models indicated a significant nonlinear relationship between WWI and CKD prevalence (nonlinear *p* < 0.05). In Model 3, this nonlinear pattern was especially prominent, exhibiting a combined U-shaped and inverted U-shaped trend (nonlinear *p* < 0.05). Specifically, WWI values within the range of 10.27 to 11.36 cm/√kg corresponded to observations of lower CKD prevalence, whereas values within 11.37 to 12.39 cm/√kg corresponded to observations of higher CKD prevalence. For WWI below 10.27 or above 12.39 cm/√kg, smaller sample sizes led to wider 95% confidence intervals, indicating greater uncertainty in prevalence estimation. The confounders adjusted for in the RCS analysis were consistent with those in logistic regression Models 2 and 3.

## Discussion

In this cross-sectional study of 5,330 adults with hyperuricemia, logistic regression models indicated a positive association between WWI and CKD, particularly among men and individuals with hyperlipidemia. To examine the nuanced relationship between WWI and CKD prevalence, RCS analysis was conducted, revealing both U-shaped and inverted U-shaped associations. These findings suggest that maintaining VAT within an optimal range is associated with a lower prevalence of CKD.

Previous studies have explored the link between VAT and CKD risk. Analysis from the Jackson Heart Study revealed that greater visceral obesity, measured via abdominal CT, was independently associated with increased CKD risk [15]. Do et al. used bioelectrical impedance analysis (BIA) and found that higher visceral fat area (VFA) correlates with elevated CKD risk, with the middle and highest VFA tertiles showing 1.368-fold and 2.027-fold increased CKD risk, respectively, compared to the lowest tertile [16]. Kramer et al., using WC as an indicator of abdominal obesity, reported that each 1 cm increase in WC was associated with a 2% increase in CKD mortality risk in fully adjusted models [17]. Collectively, these findings underscore that visceral obesity is a significant factor in renal risk. Our study aligns with this evidence. However, the methods for assessing VAT present a dilemma: CT and BIA are costly and complex, limiting their clinical utility, while conventional anthropometrics like WC and waist-to-hip ratio lack specificity [18]. To address these limitations, our study employs the WWI as a superior anthropometric indicator. WWI refines WC by incorporating body weight, providing a more specific measure of central obesity and better discriminating between adiposity and muscularity due to its positive correlation with fat mass and negative correlation with muscle mass [19, 20]. By applying this refined metric to a hyperuricemic population—a group often plagued by complex metabolic disturbances—our study addresses a critical gap in the literature regarding VAT’s role in CKD risk in this high-risk cohort.

Visceral obesity, unlike generalized obesity, involves fat accumulation around the abdominal cavity and internal organs, often characterized as metabolically unhealthy obesity (MUHO). In contrast to metabolically healthy obesity (MHO), MUHO is linked to increased insulin resistance and systemic inflammation, driven by dysregulated adipokines, which raises risks for T2DM and cardiovascular disease (CVD) [21]. The VAT-CKD relationship is further influenced by adipokines, such as adiponectin and leptin. Individuals with visceral obesity typically exhibit lower adiponectin and higher leptin levels [22]. Adiponectin alleviates oxidative stress and inflammation and activates the AMP-activated protein kinase (AMPK) pathway, providing renal protection. Leptin, in contrast, is pro-inflammatory and activates pathways like Janus Kinase 2 - Signal Transducers and Activators of Transcription (JAK2-STATs) and AMPK, promoting glomerulosclerosis and fibrosis. Blocking leptin signaling may mitigate its adverse effects on renal function [23]. These adipokines also influence β-cell function and insulin resistance, contributing to the progression of T2DM, non-alcoholic steatohepatitis, and CVD, thereby compounding renal risk [22]. Furthermore, hyperuricemia, involving dysfunction of renal and intestinal urate transporters (URAT1, GLUT9, and ABCG2), promotes renal injury and systemic metabolic dysregulation. Thus, visceral obesity and dysuricemia likely act synergistically to accelerate renal function decline [12].

Our findings also demonstrate that VAT accumulation is significantly associated with a higher prevalence of CKD in males and in individuals with hyperlipidemia, which aligns with previous research. Iseki et al. reported that abdominal obesity accompanied by metabolic abnormalities increases the incidence of CKD, with this association being more pronounced in men [24]. This gender-specific disparity in the WWI-CKD relationship may stem from distinct patterns of adipose tissue expansion. Women predominantly undergo adipocyte hyperplasia, which supports better vascularization, maintains insulin sensitivity, and preserves adipokine balance. In contrast, men are more prone to adipocyte hypertrophy, which triggers a cascade of events including local hypoxia, fibrosis, and inflammation, culminating in adipose dysfunction. This dysfunction, in turn, disrupts glucose and lipid metabolism and promotes ectopic lipid deposition [25]. This mechanistic distinction may explain the higher risk of severe kidney damage among obese men. Lipid metabolic disequilibrium-induced renal lipid accumulation represents a core pathogenic event in obesity-induced kidney injury [26]. In the context of obesity, adipose dysfunction elevates circulating free fatty acids (FFA). Among these, palmitate, a key FFA, serves as the substrate for the synthesis of ceramide—the central precursor to all complex sphingolipids—catalyzed by serine palmitoyltransferase and ceramide synthase [27]. Excess ceramide induces podocyte injury and glomerulosclerosis through inflammation and direct toxicity, while reducing sphingolipids alleviates obesity-related kidney disease [28, 29]. Additionally, hyperuricemia features systemic lipidomic disruptions, including elevated sphingolipids, and reprograms lipid metabolism by activating SREBP-1c/LXR and suppressing JAK2/STAT3 signaling, thereby promoting lipogenesis and impairing lipolysis [30, 31]. These interconnected disruptions form a vicious cycle exacerbating visceral adiposity and renal injury. In conclusion, the VAT-CKD association is particularly pronounced in men and individuals with hyperlipidemia. The distinct adipose tissue expansion patterns in men and the underlying lipid metabolic disturbances in hyperlipidemia may account for these findings, warranting further investigation into these high-risk subgroups.

Regarding the nonlinear association between WWI and CKD, our study found that CKD risk decreased when WWI values ranged between 10.27 and 11.36 cm/√kg. This aligns with findings from Scialla et al., who observed a U-shaped relationship between VAT and CKD, using CT to assess VAT volume [15]. While our study utilized WWI and predominantly included a non-Hispanic White cohort, Scialla et al. employed CT in an African American cohort. Nevertheless, the similar outcomes underscore the potential protective role of optimal VAT levels across diverse populations. Adipose tissue not only serves as an energy reserve but also acts as an endocrine and immune regulator, essential for energy homeostasis [32]. For example, adiponectin activates the AMPK pathway via AdipoR1 and the Peroxisome Proliferator-Activated Receptor Alpha (PPARα) pathway via AdipoR2, providing renal protection [33]. Adipokines generally reduce inflammation, hyperlipidemia, diabetes, and CVD risks, thereby minimizing kidney damage [23]. Adipose tissue also contains immune cells, such as macrophages and lymphocytes, which modulate immune responses [34]. However, the balance is disrupted at both extremes. Excess VAT leads to macrophage infiltration and the release of pro-inflammatory adipokines, which promote chronic inflammation, oxidative stress, and fibrosis, ultimately impairing kidney function [35]. Visceral obesity also impairs glucose and lipid metabolism, heightening risks of T2DM and CVD, which further elevates CKD risk [36]. Weight deficiency is similarly associated with an increased risk of CKD. A nationwide cohort study associated low BMI in T2DM individuals with a higher risk of end-stage renal disease, even after adjusting for confounders [37]. A 10-year cohort study also linked reductions in weight and WC to higher long-term mortality [38]. These findings highlight the significance of the association between optimal VAT distribution and kidney health, as both excessive and insufficient VAT disrupt metabolic balance, heighten inflammation, and are linked to kidney impairment.

This study has several strengths. First, we used the WWI, a metric that more accurately reflects central fat distribution, to examine the dose-response relationship between WWI and CKD prevalence in a hyperuricemic population within a cross-sectional framework. Second, the study spans multiple cycles, with a large, representative sample size. Additionally, adjusting for various key confounders enhances the robustness of the WWI-CKD association. However, this study has limitations. As a cross-sectional study, it cannot establish causality between WWI and CKD. Additionally, the absence of a universally accepted definition for hyperuricemia might affect the generalizability of our findings, as studies employing different diagnostic thresholds might identify slightly different at-risk populations. Moreover, our findings from a predominantly non-Hispanic White cohort warrant further validation in other racial and ethnic groups. Lastly, despite adjustments for known confounders, residual confounding cannot be entirely excluded due to the multifactorial nature of CKD.

## Conclusions

In conclusion, this study identifies a U-shaped and inverted U-shaped nonlinear association between the WWI and CKD among hyperuricemic adults. Specifically, with 11.36 cm/√kg observed as the turning point, WWI within the range of 10.27 to 11.36 cm/√kg was linked to a low prevalence of CKD, whereas values between 11.37 and 12.39 cm/√kg were correlated with a high prevalence. Further research is needed to confirm and better understand these associations.

## Supplementary Information

Supplementary Material 1. Fig. S1 RCS analysis of the relationship between WWI and CKD in hyperuricemic populations (Model 1 and Model 2). (a) Model 1: Unadjusted. (b) Model 2: Adjusted for age, gender, and race.

## Data Availability

All data are publicly accessible on the NHANES website (https://wwwn.cdc.gov/nchs/nhanes/).
