# Supplementary figures and images for: U- and inverted U-shaped link between weight-adjusted waist index and chronic kidney disease in hyperuricemic adults
Source: BMC Public Health. 2025 Dec 28;26:396. doi: 10.1186/s12889-025-26076-8 (PMC12859891; doi:10.1186/s12889-025-26076-8)

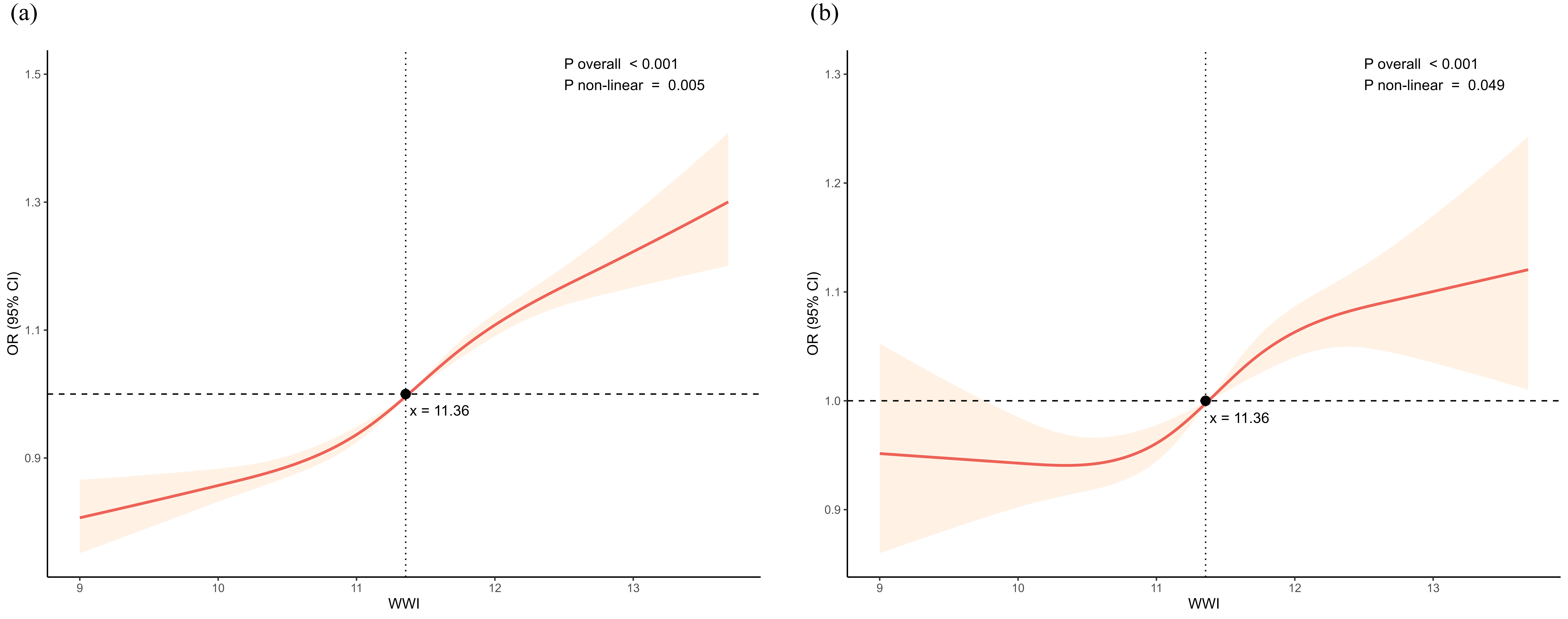

Supplement: Supplementary file 1 — Supplementary Material 1. Fig. S1 RCS analysis of the relationship between WWI and CKD in hyperuricemic populations (Model 1 and Model 2). (a) Model 1: Unadjusted. (b) Model 2: Adjusted for age, gender, and race. [file 12889_2025_26076_MOESM1_ESM.tif]
